# Integrated vector and arbovirus surveillance in Cyprus: first reports of Usutu virus and *Culex pipiens* bioform diversity highlight potential for zoonotic arbovirus transmission

**DOI:** 10.1186/s13071-026-07350-z

**Published:** 2026-03-18

**Authors:** Aayushi A. Sharma, Angeliki F. Martinou, Dániel Cadar, Michalis Omirou, Marco Neira, George K. Christophides

**Affiliations:** 1 Climate and Atmosphere Research Centre (CARE-C), The Cyprus Institute, Nicosia, Cyprus; 2 Department of Life Sciences, Imperial College London, London, SW7 2AZ UK; 3 Joint Services Health Unit, British Forces Cyprus, RAF Akrotiri, BFPO 57 Akrotiri, Cyprus; 4 Enalia Physis, Environmental Research Centre, Larnaka, Cyprus; 5 Virus Metagenomics and Evolution Group, WHO Collaborating Centre for Arbovirus and Hemorrhagic Fever Reference and Research, Bernhard Nocht Institute for Tropical Medicine, 20359 Hamburg, Germany; 6 Department of Arbovirology and Entomology, Bernhard Nocht Institute for Tropical Medicine, Hamburg, Germany; 7 Department of Agrobiotechnology, Environmental Microbiology and Biotechnology Centre, Ministry of Agricultural Rural Development and Environment, Agricultural Research Institute, Athalassa, 1516 Nicosia, Cyprus

**Keywords:** Usutu virus, *Culex pipiens molestus*, *Culex pipiens* complex, Blood meal analysis, Transmission dynamics, Vector-borne diseases, Climate change, Cyprus

## Abstract

**Background:**

Anthropogenic pressures, including urbanisation, globalisation and climate change, have facilitated an increased risk for emergence or re-emergence of mosquito-borne diseases into regions such as the Eastern Mediterranean and Middle East. Cyprus is a major stop-over site for migratory birds and has previously experienced outbreaks of West Nile virus (WNV). The island has native mosquito vector populations; however, it has also seen the recent establishment of invasive *Aedes albopictus* and *Ae. aegypti* mosquitoes. Given the dynamic climatic conditions and the shifting ecological and epidemiological landscapes in the region, the need for routine vector and pathogen surveillance has never been more critical.

**Methods:**

Herein, we present the results from localised adult mosquito surveillance that were conducted in two cities of Cyprus between 2019 and 2022. Mosquito taxa were identified through morphological analysis, and molecular techniques were used to further characterise the *Culex pipiens* bioforms. Engorged mosquito midguts were analysed to determine host blood meals. Metagenomic next-generation sequencing was employed to screen mosquito pools for arboviruses.

**Results:**

Our results provide the first report of Usutu virus in *Cx. pipiens* mosquitoes in Cyprus. Blood meal analysis identified multiple vertebrate hosts, including Cetti’s warbler, a bird species previously reported to be seropositive for WNV on the island. Additionally, we report the presence of both *Cx. pipiens pipiens* and *Cx. pipiens molestus*, an ornithophilic and a mammophilic bioform, respectively, as well as their hybrids.

**Conclusions:**

Our findings highlight the urgent need for enhanced mosquito surveillance strategies where mosquito populations will be regularly screened for pathogens to mitigate emerging risks of arbovirus transmission in Cyprus.

**Graphical abstract:**

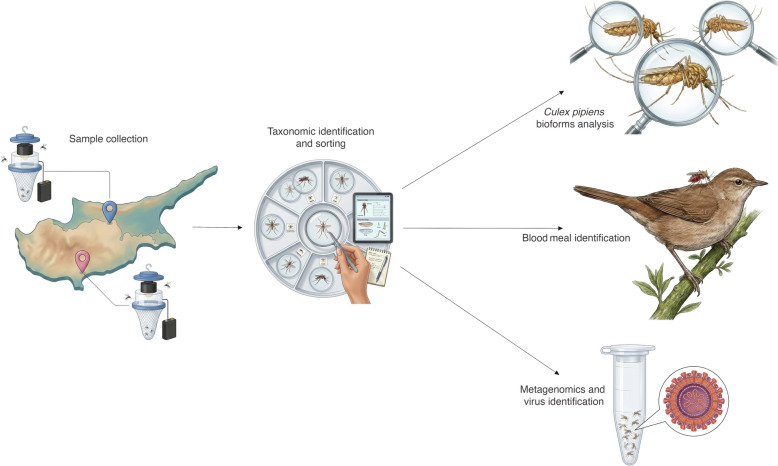

**Supplementary Information:**

The online version contains supplementary material available at 10.1186/s13071-026-07350-z.

## Background

Mosquitoes (Diptera: Culicidae) are vectors of medically important arthropod-borne viruses (arboviruses), including dengue virus (DENV), chikungunya virus (CHIKV), Zika virus (ZIKV) and West Nile virus (WNV). These pathogens pose a significant public health burden, affecting millions of people annually. The expansion of mosquito populations and associated diseases is driven by a complex interplay of climatic, ecological and socio-economic factors that interact in largely non-linear and unpredictable ways, facilitating the spread of vectors and diseases into new geographic regions [1, 2]. The Eastern Mediterranean and Middle East (EMME) region exemplifies how these factors contribute to the increasing transmission of vector-borne diseases. Anthropogenic climate change has intensified extreme weather events across the EMME, including severe heatwaves, prolonged droughts, dust storms and unprecedented air pollution [1]. Environmental models predict that these trends will persist or even accelerate in the coming decades, exacerbating conditions conducive to vector expansion and disease transmission [3]. Accordingly, predictive studies suggest that the incidence of mosquito-borne diseases in the region is likely to rise [2, 4].

Cyprus, the third-largest island in the Mediterranean and the southeastern most European Union state, is particularly vulnerable to these shifting epidemiological landscapes as it experiences warmer climate at nearly double the global average rate, alongside other environmental changes that are conducive to the emergence of new, climate-sensitive, vectors and vector-borne diseases [1–4]. A comprehensive revision of the island’s mosquito fauna published in 2009 identified 23 species [5], while more recent studies have added 3 more species, bringing the total to 26 identified species [6, 7]. These new additions include *Aedes cretinus*, a native mosquito for the Mediterranean region [8, 9], the invasive *Ae. albopictus*, which is vector of DENV, CHIKV and ZIKV globally [6, 7], as well as *Ae. aegypti*, the yellow fever mosquito which was historically recorded but had not been detected in recent decades [6]. These findings highlight the high ecological and epidemiological suitability for the establishment and spread of native and non-native mosquito vectors on the island.

Among the most abundant mosquito species in Cyprus, *Culex pipiens* is of medical and veterinary importance. It is a known vector of multiple viruses, including WNV, Rift Valley fever, Sindbis virus and Usutu virus (USUV), as well as canine dirofilarial worms and avian malaria parasites [5, 10]. *Culex pipiens* comprises two morphologically indistinguishable bioforms: *Cx. pipiens pipiens* and *Cx. pipiens molestus*, which exhibit distinct eco-ethological characteristics. *Cx. pipiens pipiens* primarily mates in open spaces, has a strong preference for feeding on birds (ornithophilic), requires a blood meal for egg development (anautogenous), and undergoes winter diapause (heterodynamic). In contrast, *Cx. pipiens molestus* mates in confined spaces without swarming, feeds predominantly on humans and other mammals (mammophilic), can reproduce without a blood meal (autogenous) and remains active all year-round. Despite their distinct eco-ethological traits, the two *Cx. pipiens* bioforms can interbreed, producing hybrids that can act as bridge vectors, facilitating virus transmission from avian reservoirs to mammalian hosts, including humans and horses [11]. In Cyprus, WNV has been detected in humans, horses, birds and most recently, mosquitoes [12–19]; however, no prior studies have confirmed the presence of *Cx. pipiens molestus* or *Cx. pipiens* hybrids in the country, despite their likely role in WNV transmission to humans and horses.

Beyond WNV, Cyprus has a broader arboviral risk landscape, with several pathogens considered either historically present or periodically introduced through travel. For example, recent assessments highlight the island’s vulnerability to DENV due to increasing human mobility [20]. Additionally, Cyprus lies along a major migratory flyway for birds travelling between Africa, Europe and Eurasia. Millions of migrating birds visit the island annually, some of which may be carrying pathogens acquired along their transcontinental routes. The proximity between migratory birds and local mosquito populations, facilitated by the island’s biodiverse ecosystems, including wetlands, coastal areas and forests, creates potential pathways for the introduction and local transmission of previously unreported viruses. Additionally, land-use changes, urban growth in proximity to wetlands and expansion of habitation into formerly uninhabited areas exacerbates the likelihood of pathogen spillover between natural habitats and urban settings. Although autochthonous transmission of arboviruses other than WNV have not been documented, given the evolving climatic conditions and the presence of competent arboviral vectors on the island, there is an urgent need to implement routine surveillance programmes aimed at monitoring pathogens within vector reservoirs and human hosts across Cyprus.

Large-scale vector-borne disease surveillance is often constrained by logistical and financial challenges. Targeted mosquito surveillance in key areas of interest offers a more efficient alternative, enabling focused resource allocation and in-depth assessments. The integration of novel molecular tools, such as metagenomic next-generation sequencing (mNGS), further enhances surveillance efforts, improving the resolution of vector population and disease dynamics, expanding our understanding of viral diversity, and detecting pathogens that might otherwise be missed in broader monitoring initiatives. Here, we present the results of a mosquito surveillance study in Cyprus that combined molecular screening to identify native and non-native mosquito species; blood meal analysis to characterise mosquito host preference; and mosquito-borne pathogen detection to assess potential risks to public and animal health. Our study provides critical insights into local vector ecology and arbovirus transmission dynamics, particularly in urban and peri-urban habitats where risk of mosquito–human contact is elevated. The findings underscore the importance of incorporating integrated surveillance tools such as pathogen screening and blood meal analysis into routine monitoring to strengthen preparedness and control strategies especially crucial in mosquito hotspots, such as large cities and wetlands undergoing urbanisation. These efforts will better inform future surveillance and control efforts in Cyprus and the wider EMME region.

## Methods

### Sampling locations and mosquito collections

**Fig. 1:**
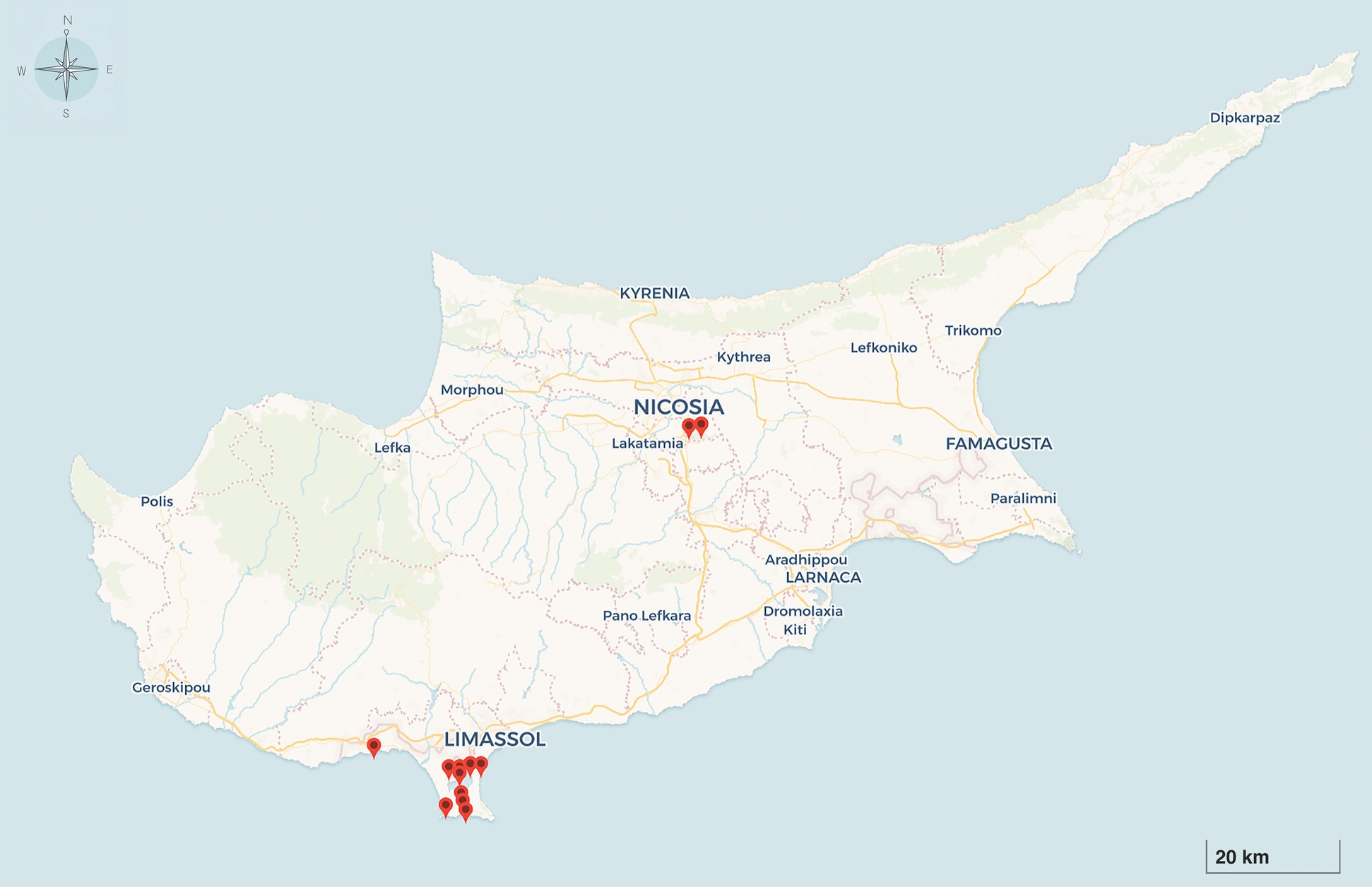
Map of Cyprus showing mosquito collection sites in Limassol and Nicosia. Red pins indicate sampling locations surveyed between May 2019 and November 2022. Map generated R using the ‘leaflet’ package with custom icons on CartoDB Voyager basemap tiles ( © CartoDB, © OpenStreetMap contributors)

Mosquito collections were conducted between November 2019 and May 2022 across 13 sampling sites located in the Limassol and Nicosia regions (Fig. 1 and Additional File 1: Supplementary Table S1). Collections in Limassol were part of a vector surveillance programme managed by the Joint Services Health Unit (JSHU) within the British Sovereign Base Areas of Cyprus. In Nicosia, collections were carried out under a surveillance initiative monitoring the presence of invasive *Aedes* mosquitoes in the urban environment within the Cyprus Institute campus, as well as at the local Athalassa Forest, a habitat regularly visited by diverse resident and migratory bird species. In 12 out of 13 locations, adult mosquitoes were collected using encephalitis virus surveillance (EVS) CO_2_ traps. At the Cyprus Institute, BG-Sentinel traps were used, while in the Athalassa Forest, Centers for Disease Control and Prevention (CDC) light traps were placed alongside EVS CO_2_ traps to maximise species diversity (Additional File 1: Supplementary Table S1). Traps were set at dawn and operated for a minimum of 12 h. Collected specimens were transported on dry ice and stored at −80 °C until further processing.

### Morphological species identification

Mosquito specimens were processed under cold conditions, and species-level taxonomic identification was performed using MosKeyTool [21]. Male specimens and those too damaged for reliable identification were excluded from downstream analyses. After identification, specimens were sorted by species, date and sampling location. Representative specimens of each identified species were preserved as voucher samples and deposited at the JSHU.

### *Cx. pipiens* bioform analysis

A subset of 300 *Cx. pipiens* specimens was selected from different locations and collection dates for bioform molecular characterisation according to a previously published protocol [22]. Briefly, genomic DNA (gDNA) was extracted using the DNeasy Blood and Tissue Kit (Qiagen, Germany), according to the manufacturer’s instructions. Bioform identification was performed using multiplex polymerase chain reaction (PCR) targeting the CQ11 microsatellite region (Additional File 1: Supplementary Table S2), with amplicons visualised on a 2% agarose gel. Differential DNA fragment amplification enabled molecular discrimination between *Cx. pipiens pipiens*, *Cx. pipiens molestus* and their hybrids. A subset of amplicons corresponding to the *Cx. pipiens pipiens* and *Cx. pipiens molestus* was subjected to Sanger sequencing (Genewiz, UK) using primers listed in Additional File 1: Supplementary Table S2. Unfortunately, owing to limited samples, Sanger sequencing could not be carried out on the target sequences of the hybrids. Resulting sequences were queried against the National Centre for Biotechnology Information (NCBI) core nucleotide database using the Basic Local Alignment Search Tool (BLAST) [23]. In cases where PCR amplification failed to produce discernible bands, a second PCR targeting nucleotide sequence differences in the acetylcholinesterase-2 (*ACE-2*) gene was performed to assess the potential presence of *Cx. quinquefasciatus* or *Cx. torrentium* as described previously [24] (Additional File 1: Supplementary Table S2). Reference DNA samples of *Cx. pipiens pipiens*, *Cx. pipiens* *molestus* and *Cx. quinquefasciatus* were used as positive controls. Negative controls consisted of nuclease-free UltraPure water (Invitrogen, UK) instead of DNA template.

### Blood meal host analysis

Mosquitoes with fully or partially engorged midguts were individually set aside following morphological identification for blood meal source analysis. gDNA was extracted using the DNeasy Blood and Tissue Kit (Qiagen, Germany), and host identification was performed by amplifying a fragment of the mitochondrial cytochrome b (*CytB*) gene, as described previously [25] (Additional File 1: Supplementary Table S2). The PCR products were visualised on a 1% agarose gel and bi-directionally sequenced. The obtained sequences were aligned against previously published GenBank sequences using BLASTn to determine the blood meal sources.

### Arboviral screening through mNGS

Mosquito specimens not used for *Culex* bioforms or blood meal analyses were sorted into pools of up to 20 individuals for arboviral screening. Pools were assembled on the basis of species, collection site and collection date. Specimens were mechanically disrupted in 1.5 mL screw-top tubes (VWR, UK) containing glass beads and 850 µL of Dulbecco’s modified Eagle’s medium (Thermo Fisher Scientific, UK). Homogenisation was performed using a TissueLyserII (Qiagen, Germany) for 10 s at 30 Hz, followed by centrifugation at 12,000 rpm for 3.5 min at 4 °C. Supernatants were filtered through 0.45 µM syringe filters (Millipore, Germany) to remove cell debris and bacteria.

A 200 µl aliquot of the filtrate was subsequently treated with a nuclease cocktail to degrade non-encapsidated nucleic acids, including residual host genetic material and bacterial contaminants. Viral RNA was then extracted using the QIAamp Viral RNA Mini Kit (Qiagen, Germany). For unbiased amplification, viral RNA was reverse transcribed with random primers and subsequently PCR amplified. The resulting complementary DNA (cDNA) was used to generate sequencing libraries with the QIAseq FX DNA Library Kit (Qiagen, Germany). Prepared libraries were quantified, normalised and multiplexed for high-throughput sequencing on the Illumina NextSeq 2000 platform using a 200-cycle paired-end protocol [2 × 100 base pair (bp) reads] with version 2.5 chemistry (Illumina, USA).

Initial raw sequence reads underwent quality control and trimming, after which assembly was performed de novo using the Trinity software (Broad Institute, USA) [26]. Using NCBI’s BLASTx platform, assembled contigs were queried against both viral reference and comprehensive non-redundant protein databases, applying an E-value threshold of 0.001 to identify viral candidates. Identified sequences were visualised and taxonomically profiled in MEGAN [27]. Genome reconstruction, open reading frame (ORF) annotation and comparative nucleotide/amino acid analyses were completed using Geneious Prime (Biomatters, New Zealand). To confirm the presence of USUV identified via mNGS, enriched viral nucleic acids were subjected to reverse transcription polymerase chain reaction (RT-PCR) for targeted virus detection [28].

### Phylogenetic analysis

To identify homologous sequences, the USUV sequence obtained from our samples was queried against NCBI’s core nucleotide database using BLAST with default parameters. Additionally, representative USUV sequences that were previously classified as belonging to the Africa 2 and 3 (AF2–3), and Europe 1–4 (EU1–4) lineages were selected on the basis of published phylogenetic classifications [29–32]. Multiple sequence alignment was performed using MUSCLE, and further trimming of sequences to improve accuracy of phylogenetic inference was carried out in MEGA11 [33, 34], followed by maximum likelihood tree construction using IQ-TREE with the ultrafast 1000 bootstrap approximation and visualisation using iTOL [35–37].

### Infection rate estimation

Infection rates (IR) were estimated using the PooledInfRate (version 4.0) add-in package for Microsoft Excel (Microsoft Corporation, USA) [38]. Following the developer’s recommendations, we used the bias-corrected maximum likelihood estimate (MLE) method to obtain the maximum likelihood estimate of the IR (MLE-IR). We selected this estimate instead of the more traditional minimum infection rate (MIR) because, in contrast to MIR, MLE-IR calculations do not assume a single positive individual per positive pool. Resulting IRs are presented as values per 1000 individuals. Confidence intervals (CI) are likelihood-based and skewness-corrected [38].

## Results

### Mosquito diversity

A total of 5904 mosquitoes were collected from 13 sampling sites across Cyprus between November 2019 and May 2022. Of these, 5801 specimens corresponded to female individuals identifiable to the species level (Additional File 1: Supplementary Table S3). A total of 28 specimens were preserved as reference samples, while the remaining 5773 were processed for downstream analyses.

Mosquitoes from nine species spanning four genera were collected and morphologically identified. *Aedes detritus* was the most abundant species, accounting for 53.21% of the total collection, followed by *Cx. pipiens* (34.67%) and *Ae. caspius* (10.69%). Other species detected included *Cx. perexiguus*, *Cx. theileri*, *Cx. brumpti*, *Culiseta* *longiareolata*, *Cs. annulata* and *Anopheles claviger*, each representing less than 5% of the total collection (Additional File 1: Supplementary Table S3). No invasive *Aedes* species (*Ae. aegypti* or *Ae. albopictus*) were detected.

**Fig. 2:**
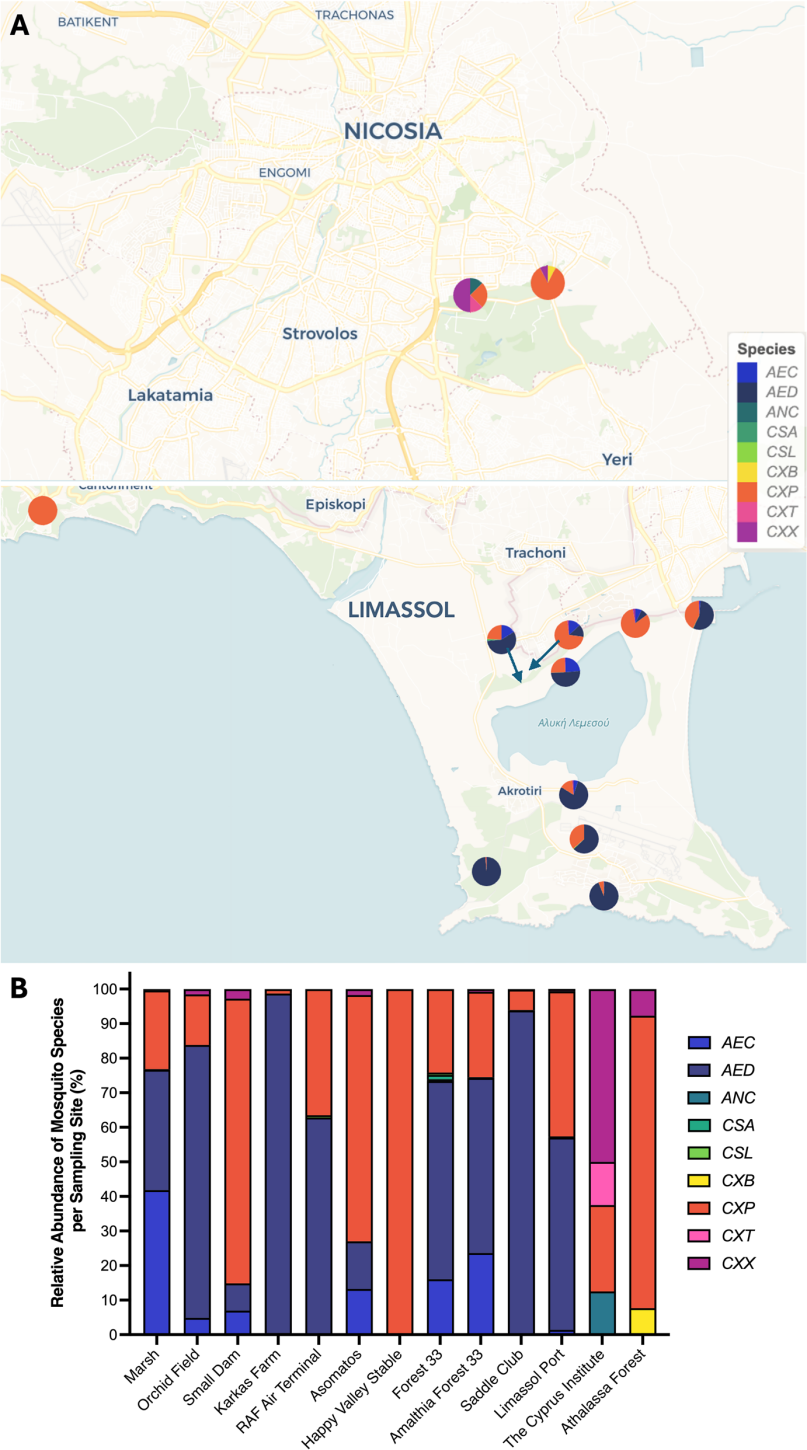
Mosquito species composition and relative abundance at each sampling site across 13 locations in Cyprus between November 2019 and May 2022. **A** Relative mosquito species abundance (%) is shown for each site using pie charts on maps of Nicosia and Limassol. Map generated in R using the ‘leaflet.minicharts’ package ( © CartoDB, © OpenStreetMap contributors). **B** A corresponding stacked bar chart provides improved visual clarity of species proportions. *AEC*, *Aedes caspius*; *AED*, *Aedes detritus*; *ANC*, *Anopheles claviger*; *CSA*, *Culiseta* *annulata*; *CSL*, *Culiseta* *longiareolata*; *CXB*, *Culex brumpti*; *CXP*, *Culex pipiens*; *CXT*, *Culex theileri*; *CXX*, *Culex perexiguus*

The highest diversity was recorded at Limassol port (seven species), Forest 33 (six species) and Amalthia Forest 33 (six species) (Fig. 2). Conversely, the lowest diversity was observed at Episkopi Happy Valley Stable, Karkas Farm, RAF Air Terminal and The Cyprus Institute, where three or fewer species were recorded. *Culex* species (*n* = 4) were found at all sampling sites. Two species, *Cx. brumpti* and *Cx. theileri*, were represented by a single specimen each. At Episkopi Happy Valley Stable, *Cx. pipiens* was the only species captured (Fig. 2 and Additional File 1: Supplementary Table S3).

**Fig. 3:**
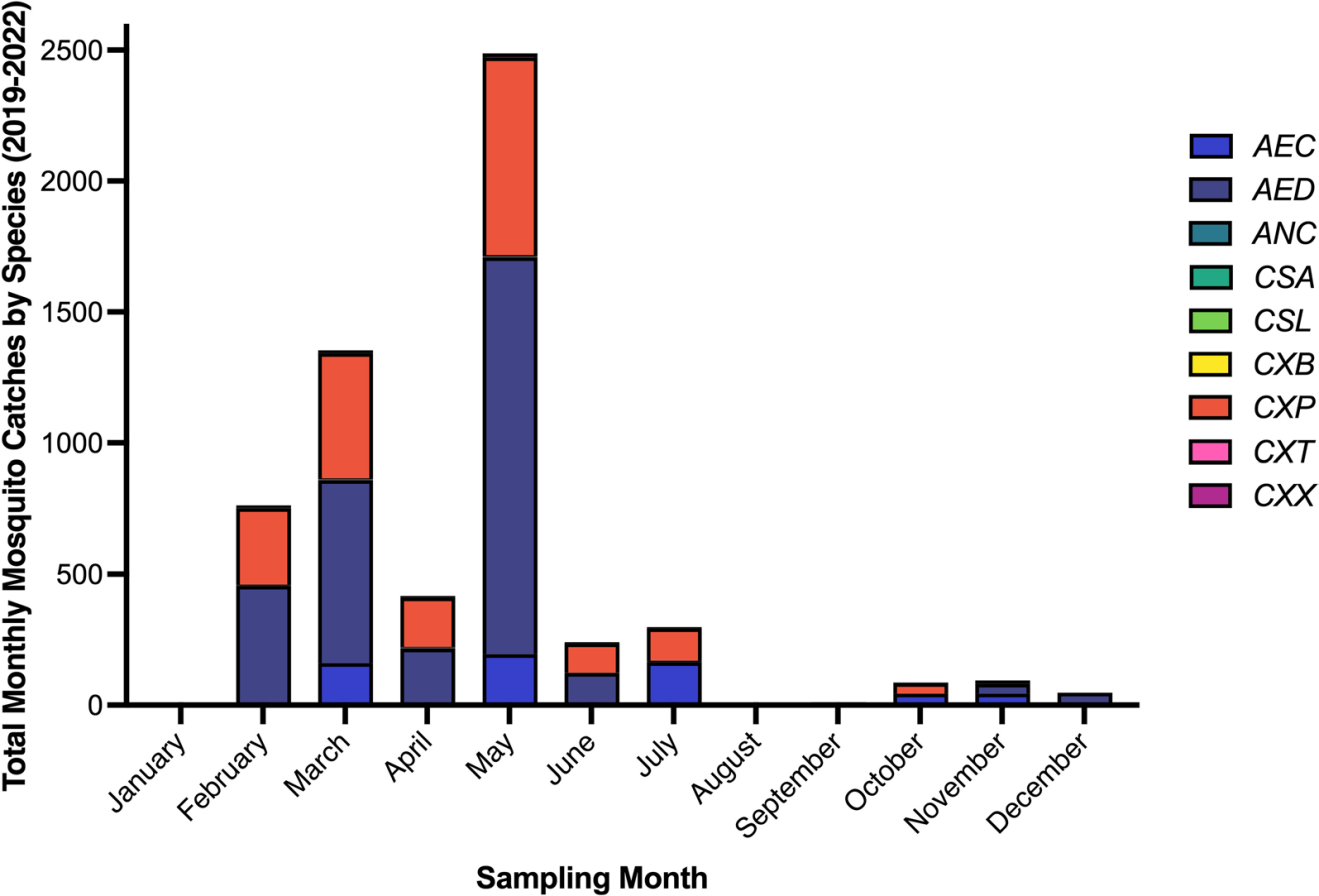
Aggregate monthly mosquito catches by species across 2019–2022. *AEC*, *Aedes caspius*; *AED*, *Aedes detritus*; *ANC*, *Anopheles claviger*; *CSA*, *Culiseta* *annulata*; *CSL*, *Culiseta* *longiareolata*; *CXB*, *Culex brumpti*; *CXP*, *Culex pipiens*; *CXT*, *Culex theileri*; *CXX*, *Culex perexiguus*

Mosquito abundance varied seasonally, with the highest numbers recorded between February and May of 2019 and 2022, while August, September and January exhibited the lowest abundance. Across all sampling periods, *Aedes* and *Culex* species accounted for most collected specimens (Fig. 3).

### Molecular characterisation of *Cx. pipiens* bioforms

**Fig. 4:**
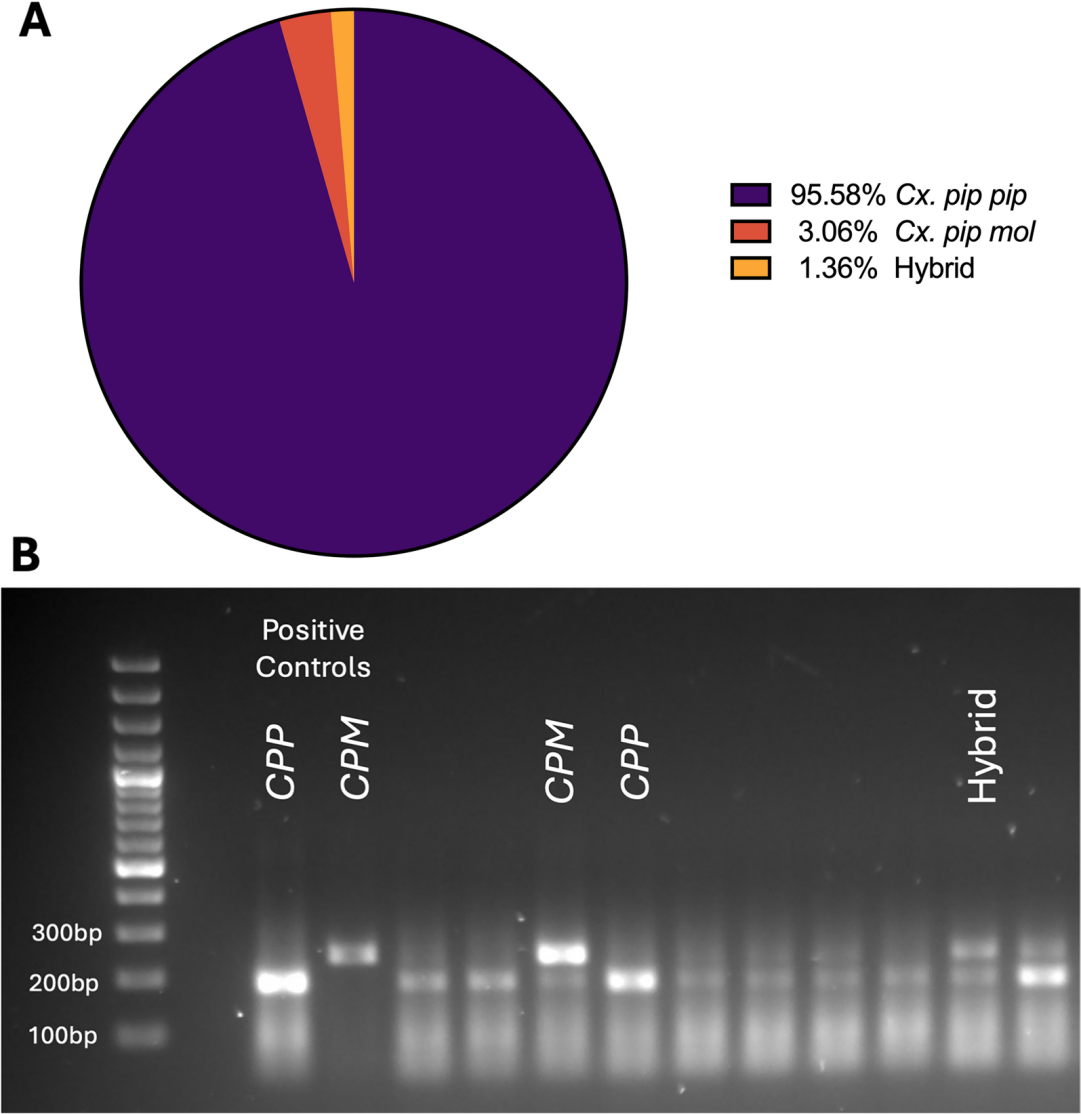
Molecular identification of *Cx. pipiens* bioforms in Cyprus. **A** Proportion of *Cx. pipiens* bioforms and their hybrids among 300 molecularly identified specimens. **B** Agarose gel showing differential amplification of the CQ11 microsatellite region. Lane 1: DNA ladder; lane 2: CPP (*Cx. pipiens pipiens*) positive control (180 bp); lane 3: CPM (*Cx. pipiens molestus*) positive control (250 bp). Both bands indicate hybrids

A total of 300 *Cx. pipiens* specimens were selected for molecular bioform identification. Among these, 95.58% (*n* = 281) were identified as *Cx. pipiens pipiens*, 3.06% (*n* = 9) as *Cx. pipiens molestus* and 1.36% (*n* = 4) as *Cx. pipiens/molestus* hybrids (Fig. 4). Six specimens failed to produce bands during PCR analysis and were not classified at the bioform level.

We performed Sanger sequencing in one representative specimen identified as *Cx. pipiens pipiens* and one specimen identified as *Cx. pipiens molestus*. The results validated the molecular characterisation, with the first specimen matching an existing *Cx. pipiens pipiens* reference sequence (GenBank accession no. PV156651), while the second specimen matched a reference sequence for *Cx. pipiens molestus* (GenBank accession no. PV156650). No *Cx. quinquefasciatus* or *Cx. torrentium* specimens were detected in this study.

### Blood meal source analysis

Fully or partially engorged female mosquitoes were analysed to determine blood meal sources. Despite repeated attempts, successful amplification and sequencing of the *CytB* gene was achieved for only 20 of 64 samples. BLAST analysis of produced *CytB* sequences against GenBank sequence entries revealed diverse blood meal sources, including domestic livestock such as cattle (*Bos taurus*), goat (*Capra hircus*) and chicken (*Gallus gallus*), as well as wild birds such as moorhen (*Gallinula chloropus*), Cetti’s warbler (*Cettia cetti)*, hooded crow (*Corvus cornix cornix*) and wood pigeon (*Columba palumbus*) (Table 1).

**Table 1:** Host blood meal sources identified from engorged mosquito midguts

Mosquito species (*n*)	Host species ID, common name	Genbank accession no.	Percentage similarity (%)
*Ae. detritus* (*n* = 2)	*Bos taurus*, cattle	OP859012.1	100
*Ae. detritus* (*n* = 4)	*Capra hircus*, goat	MK234705.1	99.53–100
*Ae. detritus* (*n* = 1)	*Ovis aries*, sheep	OM869878.1	100
*Ae. caspius* (*n* = 1)	*Gallus gallus*, chicken	OQ629489.1	100
*Ae. caspius* (*n* = 1)	*Bos taurus*, cattle	OP859012.1	100
*Cx. pipiens* (*n* = 1)	*Cettia cetti*, Cetti’s warbler	JX236380.1	99.75
*Cx. pipiens* (*n* = 1)	*Corvus cornix cornix*, hooded crow	NC_062298.1	99.76–99.77
*Cx. pipiens* (*n* = 1)	*Gallinula chloropus*, moorhen	JQ342138.1	99.05–99.73
*Cx. pipiens* (*n* = 8)	*Columba palumbus*, pigeon	MN122869.1	100

### Arboviral screening through mNGS and phylogenetic analysis

**Fig. 5:**
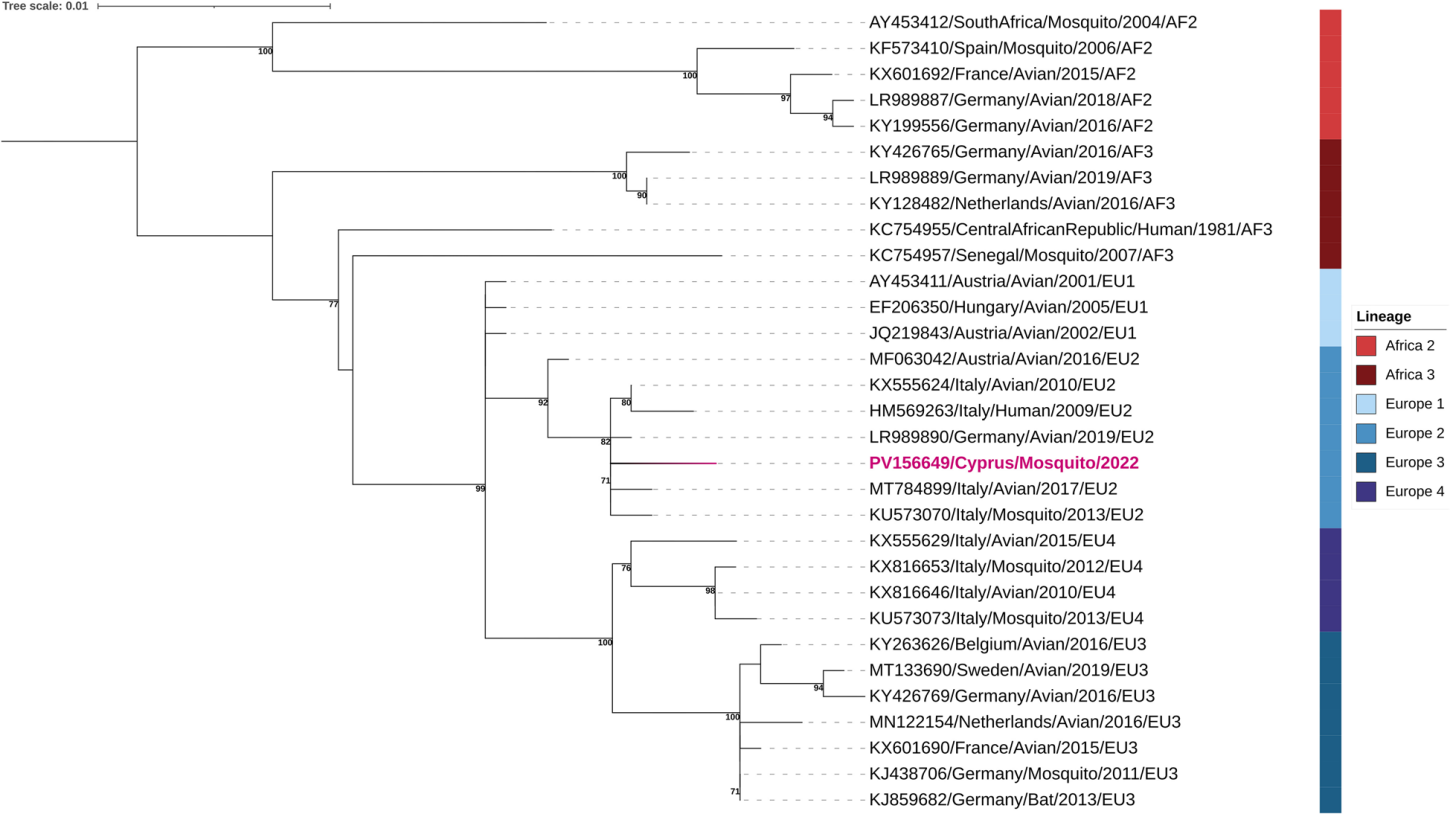
Phylogenetic analysis of the USUV Cyprus 2022 strain from a mosquito. GenBank accession no. PV156649. Sequences were aligned using MUSCLE, and maximum likelihood trees were constructed with 1000 bootstrap replications. Bootstrap support values (> 70%) are shown. Reference strains include the GenBank accession number, country, host, year of isolation and lineage. Scale bar represents nucleotide substitutions per site

A total of 5409 mosquito specimens were sorted into 343 pools for mNGS to screen for arboviruses. After quality trimming and de novo assembly, a 1134-bp contig was recovered that showed high similarity to USUV genome in BLASTx searches (GenBank accession no. PV156649). This was the only virus of known medical or veterinary relevance detected among the sequencing data. The USUV sequence was identified in a *Cx. pipiens* pool collected from the Amalthia Forest in March 2022 using an EVS CO_2_ trap operated for 24 h. The presence of USUV in the pool was further confirmed by RT-PCR [28]. Phylogenetic analysis placed the identified strain designated ‘PV156649/Cyprus/Mosquito/2022’, within the EU2 lineage of USUV, which has been associated with major European outbreaks (Fig. 5). Despite multiple attempts, viral isolation from the pool was unsuccessful owing to low viral load.

### Infection rate estimation

Because the only pool positive for USUV contained female *Cx. pipiens* samples collected during March 2022, MLE-IRs were only calculated for that species and year (Table 2). When considering all *Cx. pipiens* pools collected in Cyprus during 2022, IR was estimated at 0.86/1000 [confidence interval (CI) 0.05–4.16] (Table 2, group 1). However, if we restrict our calculations to the specific geographic area where the positive pool was collected (Amalthia Forest), the IR raises to 5.82/1000 (CI 0.35–28.28) (Table 2, group 2). In addition, for mosquitoes collected in Amalthia Forest during March 2022, the IR reached 12.04/1000 (CI 0.76–59.75) (Table 2, group 3).

**Table 2:** Infection rates (IR) of USUV in *Cx. pipiens* mosquitoes caught during 2022

Group		IR	95% CI		No. pools tested	No. positive pools	No. individuals tested
Number	Description		Lower limit	Upper limit			
1	All *Cx. pipiens* caught in Cyprus during 2022	0.86	0.05	4.16	65	1	1158
2	*Cx. pipiens* caught in Amalthia Forest during 2022	5.82	0.35	28.28	10	1	168
3	*Cx. pipiens* caught in Amalthia Forest during March 2022	12.04	0.76	59.75	5	1	79

IR were estimated using the MLE method and are expressed as infected specimens per 1000. All tested individuals are field-caught adult females

## Discussion

Given the logistical challenges associated with large-scale mosquito surveillance programmes, this study focused on location-specific monitoring of mosquitoes and arboviruses. Our aim was to assess the potential for local arboviral transmission by conducting field collections; taxonomic and molecular species identification; blood meal analysis; and molecular screening for mosquito-borne viruses.

Our results provide the first evidence of USUV in Cyprus. Phylogenetic analysis placed the strain within the EU2 lineage, which is commonly reported in European USUV outbreaks. USUV is an emerging zoonotic flavivirus within the Japanese encephalitis virus group, first isolated from *Cx. navei* mosquitoes in South Africa in 1959. It was largely overlooked as a public health threat until it was linked to significant mortality among *Turdus merula* (blackbirds) in Austria in 2001 and has caused neuroinvasive human cases in Italy (2009), Croatia (2013), the Czech Republic (2018) and Austria (2021) [39–42].

Historical sequence data suggest that USUV has been circulating in Europe since at least 1996, with its range expanding across the continent and into the Mediterranean [29]. Within the EMME region, USUV has been reported in a single pigeon in Greece [43], mosquito pools in Greece and Israel [44, 45] and human serum samples in Iraq [46]. Like WNV and other flaviviruses, USUV maintains a predominantly enzootic transmission cycle between birds and mosquitoes. However, spillover infections in humans, though rare, have been documented. Between 2012 and 2021, around 100 symptomatic human cases were reported in Europe, with 30 patients presenting neurological complications [29]. Clinical symptoms range from febrile infections to severe neuroinvasive disease [47].

USUV comprises eight phylogenetic lineages: AF1–3 and EU1–5. Notably, the EU2 lineage, detected in this study, was implicated in the 2009–2010 outbreaks in Italy, and 2016 outbreak in Austria and Hungary [29]. Given Cyprus’ location along a major migratory bird flyway between Africa and Europe, the presence of this strain is unsurprising. Further studies focusing on sequencing additional isolates will elucidate the evolutionary history and trajectory of USUV in Cyprus.

Although USUV presence was only detected in a single year and location in our study, the estimated IRs (Table 2) are within the range reported in other European regions where this virus is found. For example, surveys in Italy have reported annual USUV IRs in *Cx. pipiens* ranging from 0.24 to 25, depending on the location [48, 49]; and in Germany, only 1 positive pool was reported among 215 pools of *Cx. pipiens* tested in 2010 (corresponding to 5383 individuals) [50]. Therefore, although our data suggest low USUV IRs in mosquitoes in Cyprus, it is important to consider these results in the context of the limited chronologic and geographic coverage of our field collections. To fully understand the epidemiological landscape of this virus in Cyprus, further studies should be performed to map its geographic distribution on the island and characterise its activity periods.

Morphological identification of the field-collected specimens confirmed the presence of several previously recorded mosquito species in Cyprus (Additional File 1: Supplementary Table S3), as well as one new record: *Cx. brumpti*, increasing the mosquito fauna of the country to 27 identified species. Little is known about the biology of this species, which has been reported in Algeria, France, Italy, Morocco and Spain [51]. Unfortunately, owing to the lack of publicly available genomic data for this species, it was not possible to perform molecular confirmation of this record.

Consistent with previous studies, *Ae. detritus*, *Cx. pipiens* and *Ae. caspius* were the most abundant species in our collections [5, 6, 52]. However, it is important to note that the observed differences in captured species richness and relative abundance (Figs. 2 and 3) are likely influenced by the specific trap types deployed at each location, which have known sampling biases (e.g., CO*2*-baited traps tend to over-represent host-seeking females, whereas BG-Sentinel traps are relatively more attractive to *Aedes* spp.). Accordingly, apparent differences between sites may partly reflect sampling methodology rather than true ecological variation. The objective of our study was to maximise taxonomic coverage for pathogen detection; therefore, these figures are presented as site-level descriptive summaries rather than directly comparable estimates, and cross-site contrasts should therefore be interpreted with caution.

In Europe, *Cx. pipiens* is the primary vector of USUV, with blackbirds serving as the primary hosts. However, *Ae. albopictus*, *Ae. caspius* and *Ae. detritus* have also been implicated in USUV transmission in Italy [53]. Given that these mosquitoes are among the most common species in Cyprus, our work indicates potential for autochthonous arbovirus transmission under favourable environmental conditions and expanding urban areas into wetlands [54]. Additionally, while most blackbirds in Cyprus are passage migrants, some are resident breeders, further enhancing the risk of sustained USUV transmission [55].

This study provides the first publicly available molecular data, confirming the presence of the *Cx. pipiens molestus* bioform in Cyprus. Although the *Cx. pipiens molestus* and *Cx. pipiens pipiens*/*molestus* hybrids were detected at lower frequencies than *Cx. pipiens pipiens*, their presence carries significant public health implications owing to their mammophilic feeding behaviour. Unlike the ornithophilic *Cx. pipiens pipiens*, *Cx. pipiens molestus* readily feeds on humans and remains active year-round, making it a more efficient human vector for zoonotic viruses such as WNV and USUV. The detection of *Cx. pipiens pipiens/molestus* hybrids suggests the potential for bridge transmission, facilitating spillover events from birds to humans.

Despite a limited sample size, blood meal analysis provided key insights into mosquito host preference patterns. Successfully sequenced samples identified a diverse range of vertebrate hosts, including domestic livestock as well as multiple wild birds (Table 1). No mixed blood meals were detected. Notably, a *Cx. pipiens pipiens* specimen was found to have fed on Cetti’s warbler, a bird previously identified as WNV-seropositive in Cyprus [18]. This finding indicates potential enzootic circulation, reinforcing concerns about local WNV transmission; however, the extent of local transmission requires further investigation. Moreover, the presence of the bridge vectors, *Cx. pipiens pipiens*/*molestus* hybrids, highlights a potential for spillover transmission to humans and for autochthonous transmission of WNV on the island, warranting continued surveillance.

To strengthen these findings, further studies should incorporate alternative mosquito-trapping methods, such as gravid traps, which preferentially sample blood-fed females. Combining this approach with EVS CO_2_ traps, which predominantly attract host-seeking mosquitoes, would improve our ability to assess transmission dynamics. Furthermore, we acknowledge that our blood meal analysis had a low identification success (20/64 engorged females; 31.25%), which constrains the strength of our inferences about host use. Failed amplifications in blood-fed mosquitoes are plausibly attributable to factors such as advanced blood digestion (and consequent DNA degradation) in the mosquito’s gut, or co-extracted PCR inhibitors such as heme from midgut contents. Therefore, the successfully identified subset may be biased towards more recent feeds and hosts whose DNA persists longer; accordingly, our host-feeding results should be interpreted as indicative rather than quantitative. Additionally, the limited quantity of hybrid *Cx. pipiens* mosquitoes meant that available material had to be prioritised for diagnostic confirmation using PCR. In the future, such work would benefit from targeted sequencing to provide further valuable insight into hybrid genetic composition.

The evolving arbovirus landscape in Cyprus highlights the increasing risk of emerging mosquito-borne viruses. Our findings provide the first evidence of the presence of USUV and critical bridge vectors of this virus in Cyprus. While the prevalence of USUV appears relatively low on the basis of this initial investigation, these results establish a new and important baseline for arboviral risk on the island. They underscore the need for systematic, longitudinally designed surveillance to accurately quantify transmission intensity; delineate the geographic range of both the virus and its vectors; and inform the development of targeted, evidence-based public health interventions. Similarly, the presence of both bioforms of *Cx. pipiens* (plus their hybrids); the identification of vector and reservoir species associated with the WNV transmission cycle; and prior reports of human WNV outbreaks, collectively highlight the current risk of WNV circulation and lend support to recent calls for the implementation of enhanced monitoring, control and public information campaigns in Cyprus [20].

The detection of USUV through viral mNGS in our work highlights the value of such advanced technologies in identifying pathogens that may otherwise evade detection using conventional molecular techniques. Such tools will be instrumental in enhancing arbovirus surveillance and expanding our capacity for assessing the risk of mosquito-borne disease transmission in the region.

## Conclusions

Understanding arbovirus transmission cycles is fundamental to developing and implementing effective vector control strategies. While efforts to monitor invasive *Aedes* mosquitoes remain crucial, it is equally important to recognise the role of already established mosquito species in local arbovirus transmission. Localised studies, such as this, provide valuable insights that inform evidence-based policies and targeted public health interventions. However, with the increasing risk of arbovirus transmission due to climate change, vector range expansion and intense human mobility, there is a pressing need to implement integrative public health tools for early warning and decision support at the national and regional levels.

## Supplementary Information

**Additional file 1. Table S1**. Sampling site information including coordinates, traps used, and habitat description. Table S2. Primers used in PCR-based assays for *Cx. pipiens* bioform characterisation and bloodmeal identification. Table S3. Composition and abundance of mosquito species across sampling sites.

## Data Availability

All data generated or analysed during the current study are available in the published article and in the supplementary information files.

## Abbreviations

Arboviruses: Arthropod-borne viruses
ACE-2: Acetylcholinesterase-2
AF2-3: Africa 2 and 3
BLAST: Basic Local Alignment Search Tool
CHIKV: Chikungunya virus
CI: Confidence intervals
*CytB*: Cytochrome b
DENV: Dengue virus
EMME: Eastern Mediterranean and Middle East
EU1–4: Europe 1–4
gDNA: Genomic DNA
IR: Infection rates
JSHU: Joint Services Health Unit
MIR: Minimum infection rate
MLE: Maximum likelihood estimate
MLE-IR: Maximum likelihood estimate of the infection rate
mNGS: Metagenomic next-generation sequencing
NCBI: National Centre for Biotechnology Information
ORF: Open reading frame
USUV: Usutu virus
WNV: West Nile virus
ZIKV: Zika virus
