# Supplementary material for: Integrated vector and arbovirus surveillance in Cyprus: first reports of Usutu virus and Culex pipiens bioform diversity highlight potential for zoonotic arbovirus transmission
Source: Parasit Vectors. 2026 Mar 18;19:184. doi: 10.1186/s13071-026-07350-z (PMC13123236; doi:10.1186/s13071-026-07350-z)
Supplement: Supplementary file 1 — Additional file 1. Table S1. Sampling site information including coordinates, traps used, and habitat description. Table S2. Primers used in PCR-based assays for Cx. pipiens bioform characterisation and bloodmeal identification. Table S3. Composition and abundance of mosquito species across sampling sites. [file 13071_2026_7350_MOESM1_ESM.docx]

**Supplementary Information**

**Table S1. Sampling site information** **including coordinates, traps used, and habitat description.**

| Sampling Sites | Coordinates  (Latitude, Longitude) | Traps Used | Habitat Description |
| --- | --- | --- | --- |
| Marsh | 34.63572, 32.92114 | EVS CO_2_ | Wetland |
| Orchid Field | 34.59986, 32.97211 | EVS CO_2_ | Wetland |
| Small Dam | 34.64152, 32.99013 | EVS CO_2_ | Urban |
| Karkas Farm | 34.58117, 32.94632 | EVS CO_2_ | Urban |
| RAF Air Terminal | 34.58912, 32.97507 | EVS CO_2_ | Urban |
| Asomatos | 34.633166, 32.960367 | EVS CO_2_ | Urban |
| Episkopi Happy Valley Stable | 34.668806, 32.815472 | EVS CO_2_ | Urban |
| Forest 33 | 34.62955, 32.96073 | EVS CO_2_ | Forest |
| Amalthia Forest 33 | 34.631954, 32.962931 | EVS CO_2_ | Forest |
| Saddle Club | 34.57523, 32.98091 | EVS CO_2_ | Urban |
| Limassol Port | 34.643369, 33.00894 | EVS CO_2_ | Urban |
| Athalassa Forest | 35.143194, 33.403028  35.142861, 33.403194 | EVS CO_2_;  CDC Light | Forest |
| The Cyprus Institute | 35.14142, 33.38118 | BG-Sentinel | Urban |

**Table S2. Primers used in PCR-based assays for *Cx. pipiens* bioform characterisation and bloodmeal identification.**

| Target Gene | Species ID | Band Size (bp) | Primer Name | Primers | Reference |
| --- | --- | --- | --- | --- | --- |
| *CQ11* | Cx. pipiens molestus | 250 | CQ11F2 | GATCCTAGCAAGCGAGAAC | [21] |
|  |  |  | molCQ11R | CCCTCCAGTAAGGTATCAAC | [21] |
|  | Cx. pipiens pipiens | 180 | CQ11F2 | GATCCTAGCAAGCGAGAAC | [21] |
|  |  |  | pipCQ11R | CATGTTGAGCTTCGGTGAA | [21] |
| ACE-2 | Cx. pipiens | 610 | ACEpip | GGAAACAACGACGTATGTACT | [23] |
|  |  |  | B1246 | TGGAGCCTCCTCTTCACGGC | [23] |
|  | Cx. quinquefasciatus | 274 | ACEquin | CCTTCTTGAATGGCTGTGGCA | [23] |
|  |  |  | B1246 | TGGAGCCTCCTCTTCACGGC | [23] |
|  | *Cx. torrentium* | 416 | ACEtorr | TGCCTGTGCTACCAGTGATGTT | [23] |
|  |  |  | B1246 | TGGAGCCTCCTCTTCACGGC | [23] |
| *CytB* | Vertebrates | 457 | Cyt *b* (f) | GAGGMCAAATATCATTCTGAGG | [24] |
|  |  |  | Cyt *b* (r) | TAGGGCVAGGACTCCTCCTAGT | [24] |

**Table S3. Composition and abundance of mosquito species across sampling sites.**

|  | | Mosquito Abundance per Sampling Site (Number) | | | | | | | | |  |
| --- | --- | --- | --- | --- | --- | --- | --- | --- | --- | --- | --- |
| City | Sampling Sites | *AEC* | *AED* | *ANC* | *CSA* | *CSL* | *CXB* | *CXP* | CXT | *CXX* | Number of Specimens |
| Limassol | Marsh | 217 | 181 |  |  | 1 |  | 118 |  | 2 | 519 |
|  | Orchid Field | 16 | 259 |  |  |  |  | 48 |  | 5 | 328 |
|  | Small Dam | 66 | 75 |  |  |  |  | 782 |  | 26 | 949 |
|  | Karkas Farm | 2 | 378 |  |  |  |  | 5 |  |  | 385 |
|  | RAF Air Terminal |  | 93 |  |  | 1 |  | 54 |  |  | 148 |
|  | Asomatos | 23 | 24 |  |  |  |  | 124 |  | 3 | 174 |
|  | Episkopi Happy Valley Stable |  |  |  |  |  |  | 11 |  |  | 11 |
|  | Forest 33 | 97 | 347 | 3 | 8 | 4 |  | 146 |  |  | 605 |
|  | Amalthia Forest 33 | 180 | 386 | 1 | 1 |  |  | 188 |  | 6 | 762 |
|  | Saddle Club | 3 | 711 |  | 1 |  |  | 45 |  | 1 | 761 |
|  | Limassol Port | 16 | 633 |  | 2 | 2 |  | 477 | 1 | 7 | 1138 |
| Nicosia | Athalassa Forest |  |  | 1 |  |  |  | 2 | 1 | 4 | 8 |
|  | The Cyprus Institute |  |  |  |  |  | 1 | 11 |  | 1 | 13 |
| Total no. of specimens per species | | 620 | 3,087 | 5 | 12 | 8 | 1 | 2,011 | 2 | 55 | 5,801 |
| Mosquito abundance per species (%) | | 10.7 | 53.2 | 0.1 | 0.2 | 0.1 | 0.0 | 34.7 | 0.0 | 0.9 |  |

*AEC*, *Aedes caspius*; *AED*, *Aedes detritus*; *ANC*, *Anopheles claviger*; *CSA*, *Culiseta* *annulata*; *CSL*, *Culiseta longiareolata*; *CXB*, *Culex brumpti*; *CXP*, *Culex pipiens*; *CXT*, *Culex theileri* and *CXX*, *Culex perexiguus*
